# Prevalence of gestational diabetes mellitus and associated factors among women receiving antenatal care at a tertiary hospital in South-Western Uganda

**DOI:** 10.11604/pamj.2023.46.50.38355

**Published:** 2023-10-04

**Authors:** Irene Kahimakazi, Yarine Fajardo Tornes, Leevan Tibaijuka, Hamson Kanyesigye, Joshua Kiptoo, Musa Kayondo, Joseph Ngonzi, Kwame Adu-Bonsaffoh, Lenard Abesiga, Henry Mark Lugobe

**Affiliations:** 1 Department of Obstetrics and Gynaecology, Mbarara University of Science and Technology, Mbarara, Uganda,; 2 Department of Obstetrics and Gynaecology, Mbarara Regional Referral Hospital, Mbarara, Uganda,; 3 Department of Pharmacy, Clinical Pharmacy Unit, Mbarara University of Science and Technology, Mbarara, Uganda,; 4 Department of Obstetrics and Gynaecology, University of Ghana Medical School, Accra, Ghana

**Keywords:** Gestational diabetes, antenatal care, Mbarara, pregnancy

## Abstract

Introduction: gestational diabetes mellitus is one of the major causes of morbidity and mortality among pregnant women worldwide. We aimed to determine the prevalence and factors associated with gestational diabetes mellitus among women attending the antenatal care clinic at a tertiary care hospital in South-Western Uganda.

Methods: this was a hospital-based cross-sectional study conducted among women at ≥24 weeks of amenorrhea attending the antenatal care clinic at Mbarara Regional Referral Hospital between December 2020 and March 2021. We screened all women for gestational diabetes mellitus using the World Health Organization 2013 diagnostic criteria. We obtained socio-demographic, medical, and obstetric data. Multivariable logistic regression was used to determine the factors independently associated with gestational diabetes mellitus.

Results: we enrolled 343 pregnant women with a mean age of 27.3 (SD ±12.3) years. Of the 343 participants, 35 (10.2%) had gestational diabetes mellitus (GDM) (95% C.I: 7.4%-13.9%) and 7 (2%) had diabetes in pregnancy. The factors significantly associated with gestational diabetes mellitus were; previous history of foetal macrosomia in any of the previous pregnancies (aOR: 5.53, 95% C.I: 1.29-23.65) and family history of diabetes mellitus in the first-degree relatives (aOR: 4.45, 95% **C.I:1.48-13.34)**.

Conclusion: one in every ten pregnant women attending the antenatal care clinic at Mbarara Regional Referral Hospital is likely to have gestational diabetes mellitus in pregnancy. There is a need to strengthen routine testing for gestational diabetes mellitus among women attending the antenatal care clinic, especially pregnant women with a prior history of foetal macrosomia and a family history of diabetes mellitus in first-degree relatives.

## Introduction

Gestational diabetes mellitus (GDM) is defined as carbohydrate intolerance of variable severity with onset or first recognition during the second trimester or third trimester of the present pregnancy [1-3]. The prevalence of GDM varies across populations with a recent systematic review stating a pooled global prevalence of 10.13% (2.075% to 38.25%) [4], 9%-13.6% in Africa [5,6], 6% in East Africa [5] and 15.6% - 30.3% in health facilities in Uganda [7,8].

Gestational diabetes mellitus is associated with adverse maternal and perinatal outcomes and risk of developing diabetes and cardiovascular diseases later in life [9-11]. Early identification of women with GDM reduces their risk for adverse pregnancy outcomes [3]. It is therefore recommended that all pregnant women attending antenatal care are routinely screened for GDM [3,12]. However, universal screening is still poor in low and middle-income countries and may not be feasible due to context-specific challenges in different settings [13]. Resource limitations including cost implication preclude universal screening [13]. Given the burden and the potential maternal-perinatal complications of GDM, the identification of relevant associated clinical factors can potentially result in risk stratification and targeted screening, early diagnosis, and initiation of treatment.

A systematic review and meta-analysis documenting the burden of GDM and associated factors in sub-Saharan Africa based on the updated WHO 2013 diagnostic criteria showed that there is a paucity of studies from Uganda [5]. The studies describing the burden of gestational diabetes mellitus in Uganda have been mainly among urban populations [8] while the other used random blood sugar as one of the diagnostic tools for GDM [7]. Screening for GDM using the oral glucose tolerance test in the antenatal clinic which is the recommended test in pregnancy is not routinely done in resource limited settings [13]. We therefore sought to determine the prevalence and factors associated with GDM among women attending the antenatal clinic at Mbarara Regional Referral Hospital, South-Western Uganda.

## Methods

**Study design and site:** this was a cross-sectional study conducted at the antenatal care clinic of Mbarara Regional Referral Hospital (MRRH) from December, 2020 to March, 2021. MRRH is located in Mbarara City in South-Western Uganda and is a tertiary public hospital that serves as a teaching hospital for Mbarara University of Science and Technology. The clinic attends to an estimated 4,700 pregnant women annually.

**Study participants:** we included all pregnant women ≥24 weeks of gestation attending the antenatal care clinic at Mbarara Regional Referral Hospital who had had at least an 8-hour fast prior to data collection. We excluded women with known pre-existing diabetes mellitus and those who were not sure of the period of fasting.

**Sample size and sampling:** the sample size was determined using OpenEpi version 3 [14]. We assumed a prevalence of 27.9% GDM [15], at 95% confidence interval, 5% margin of error, and 10% non-response rate. We obtained a total sample size of 343 pregnant women. We enrolled pregnant women who met the inclusion criteria into the study at the ANC clinic through consecutive sampling.

**Data collection and laboratory procedures:** all pregnant women attending ANC were assessed for eligibility. Data were collected by trained research assistants using an interviewer-administered questionnaire. Capillary blood samples were collected from the anterolateral aspect of the pulp of the middle finger of the non-dominant hand for fasting blood glucose level testing and OGTT. The initial blood drop was wiped away with a sterile dry piece of cotton and the sample collected from subsequent blood drops onto the glucose strip fitted into a glucometer (One Touch-Select Simple Meter, in mmol/L, 10-44 o C, System Accuracy of 99.7%, SN SAGVTNLP, CR 2032, AW 06817201A, Rev. Date: 07/2012, Made in China). Participants with fasting blood sugar (FBS) <5.1 mmol proceeded to have their oral glucose tolerance test (OGTT) done. For OGGT, the participant was given a solution containing 75 g of D-glucose monohydrate-anhydrous glucose (pure glucose, Excel Chemicals Ltd, food division, made in Kenya) measured by pelouze machine and dissolved in 300 mls of drinking water. This was taken slowly over 15 minutes using a disposable cup. A timer clock was set at 2 hours. The pregnant woman was requested to sit, not to eat or drink, not to exercise, not to smoke or leave the hospital until the 2 hours were completed. At 2 hours following a 75g glucose load, the second capillary blood sample was withdrawn and results were read at 5 seconds using a One Touch Select Simple Glucometer and recorded.

The oral glucose tolerance test was done if a pregnant woman´s fasting blood glucose was <5.1 mmol/l. The participant was normoglycemic if her fasting blood glucose was < 5.1 mmol/l and a 2-hour plasma glucose were < 8.5 mmol/l following a 75 g oral glucose load, diabetes in pregnancy if her fasting blood glucose was ≥ 7.0 mmol/l or 2-hour blood glucose was ≥ 11.1 mmol/l and gestational diabetes mellitus was diagnosed if her fasting blood glucose was ≥ 5.1-6.9 mmol/l or 2-hour plasma glucose was ≥ 8.5-11.0 mmol/l. However, if a pregnant woman´s fasting blood glucose was ≥ 7.0 mmol/l, oral glucose tolerance test was not done since she was already considered to have diabetes mellitus in pregnancy [3].

**Study variables:** our primary outcome was gestational diabetes mellitus, defined as fasting blood glucose level of ≥ 5.1-6.9 mmol/l or a two-hour glucose level of ≥ 8.5-11.0 mmol/l following a 75g oral glucose load in a pregnant woman ≥ 24 weeks of gestation according to the World Health Organization 2013 diagnostic criteria [3].

The independent variables included socio-demographic, behavioral/lifestyle, obstetric and medical factors. The socio-demographics included age, residence, occupation, and level of education. Behavioural/lifestyle factors included alcohol use, smoking, and physical activity which was defined as moderate-intensity activity of 30 minutes daily translating to 150 minutes per week using the UK Chief Medical Officers Physical Activity Guidelines [16] and dietary diversity assessed using a 24-h food recall method by the Food and Nutrition Technical Assistance (FANTA) 2016 version, woman´s minimum dietary diversity measurement tool and the minimum dietary diversity score (MDDS) dichotomized on the basis of whether or not a pregnant women had consumed the list of defined food groups the previous day or night. The MDDS of five and more (≥ 5) was categorized as adequate dietary diversity and < 5 scores were categorised as inadequate dietary diversity [17]. Obstetric factors included gravidity, gestational age, history of macrosomia (defined as birth weight ≥ 4.0 kg in any of the previous deliveries), previous history of gestational diabetes mellitus, history of abortion, history of unexplained stillbirth, history of preterm birth and history of caesarean section delivery. Medical factors included chronic hypertension, family history of diabetes in the first-degree relatives (defined as immediate family members including parents, brothers, and sisters), and body mass index (BMI) ≥ 30 kg/m^2^.

**Data management and analysis:** data were entered into REDCap electronic data capture tools hosted at Mbarara University of Science and Technology (MUST) Department of Obstetrics and Gynaecology (OB/GYN) and exported to STATA version 15 (StataCorp, College Station, Texas, USA) for analysis. We described the demographic, obstetric, and medical characteristics among the study participants, and expressed the descriptive statistics as frequencies/percentages. We then compared categorical variables between women with gestational diabetes mellitus (GDM) and those without GDM using Pearson´s chi square test. The prevalence of GDM was determined as a proportion of pregnant women diagnosed with GDM, expressed as a percentage. To identify the factors associated with GDM, we used univariate and multivariable logistic regression analysis. Corresponding odds ratios (ORs) with their 95% confidence intervals (CIs) were reported. All variables associated with GDM at univariate analysis with p-value < 0.2 and those with biological plausibility were included into the final multivariable regression model, to determine the adjusted correlates of GDM. Variables with p-values < 0.05 in the final model were considered statistically significant.

**Ethical considerations:** this study was approved by the Mbarara University of Science and Technology Research and Ethics Committee (Reference Number: MUREC-17/11-20), Mbarara Regional Referral Hospital administration, and the Uganda National Council for Science and Technology (UNCST-SS854ES). All study participants provided informed written consent to participate in the study. Pregnant women who were diagnosed with gestational diabetes mellitus or diabetes mellitus during the study received immediate treatment including appropriate counselling, hypoglycaemic medications, and referral to diabetologists.

## Results

**Figure 1:**
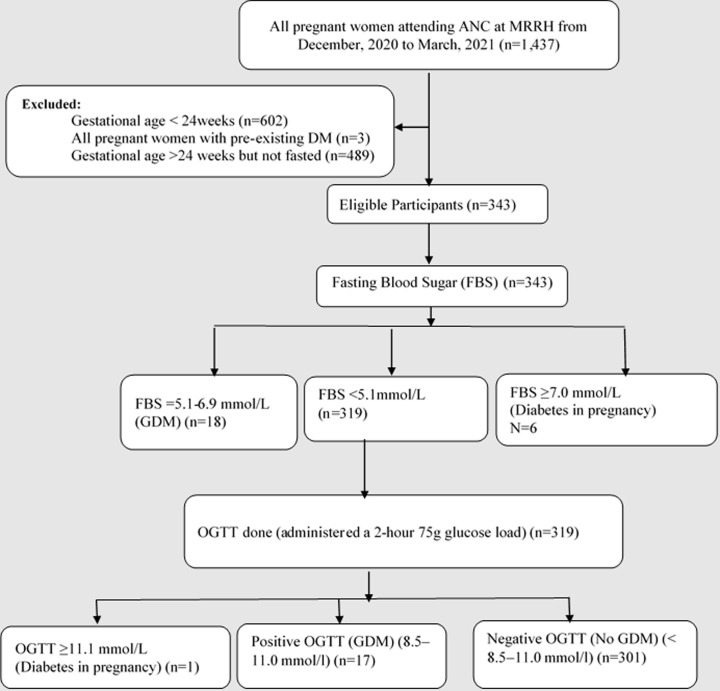
participant recruitment flow-chart

There were 1,437 pregnant women attending the antenatal care clinic from December 15, 2020 to March 15, 2021 at MRRH. Of these, 835 women had gestational age ≥ 24 weeks and 489 had not fasted for at least 8 hours while 3 had pre-existing diabetes mellitus and were therefore excluded from the study. We therefore enrolled 343 participants into our study as shown in Figure 1. Of the 343 participants, 35 had GDM, with a prevalence of 10.2% (95% CI: 7.4-13.9). Seven participants (2%) were diagnosed with diabetes in pregnancy.

**Socio-demographic and behavioural characteristics of study participants:** the mean age of the participants was 27.3 ± 12.3 years. The majority of the participants 283 (84.2%) were aged 20-34 years, had attended secondary school education 122 (36.3%), were married 330 (98.2%) and were unemployed 183 (54.5%) as shown in Table 1.

**Table 1:** socio-demographic and behavioural characteristics of pregnant women attending the antenatal care clinic (ANC) clinic at the Mbarara Regional Referral Hospital

Variable	Category	Total (N=336) N (%)	GDM status n (%)		P value
			Yes	No	
Age (years)	<20	37 (11.0)	8 (22.9)	29 (9.6)	0.057
	20-34	283 (84.2)	26 (74.3)	257 (85.4)	
	>34	16 (4.8)	1 (2.9)	15 (5.0)	
Residence	Urban	285 (84.8)	30 (85.7)	255 (84.7)	0.876
	Rural	51 (15.2)	5 (14.3)	46 (15.3)	
Level of education	Uneducated	4 (1.2)	1 (2.9)	3 (1.0)	0.286
	Primary	110 (32.7)	14 (40.0)	96 (31.9)	
	Secondary	122 (36.3)	14 (40.0)	108 (35.9)	
	Tertiary	100 (29.8)	6 (17.1)	94 (31.2)	
Marital status	Married	330 (98.2)	35 (100.0)	295 (98.0)	0.399
	Unmarried	6 (1.8)	0 (0.0)	6 (2.0)	
Employment status	Employed (formal)	153 (45.5)	19 (54.3)	134 (44.5)	0.272
	Unemployed	183 (54.5)	16 (45.8)	167 (55.5)	
Religion	Christian	307 (91.4)	29 (82.9)	278 (92.4)	0.058
	Moslem	29 (8.6)	6 (17.1)	23 (7.6)	
Duration of exercise/day (minutes)	≥ 30	301 (89.6)	30 (85.7)	268 (89.0)	0.251
	< 30	35 (10.4)	5 (14.3)	33 (11.0)	
Dietary diversity	Adequate (<5)	182 (54.2)	21 (60.0)	161 (53.5)	0.464
	Inadequate (≥5)	154 (45.8)	14 (40.0)	140 (46.5)	

Majority of the participants were multigravida 191 (56.9%), had no previous history of foetal macrosomia 214 (93.9%), were overweight 144 (42.9%), no chronic hypertension 332 (98.8%) and had no history of DM in the first-degree relatives 294 (87.5%) as shown in Table 2.

**Table 2:** obstetric and medical characteristics of pregnant women attending the antenatal care clinic (ANC) clinic at the Mbarara Regional Referral Hospital

Variable	Category	Total (N=336) N (%)	GDM status n (%)		P value
			Yes	No	
Gravidity	≤1	108 (32.1)	6 (17.1)	102 (33.9)	0.020
	2-4	191 (56.9)	21 (60.0)	170 (56.5)	
	≥5	37 (11.0)	8 (22.9)	29 (9.6)	
Gestational age (weeks)	< 28	125 (37.2)	15 (42.9)	110 (36.5)	0.690
	28-37	157 (46.7)	14 (40.0)	143 (47.5)	
	> 37	54 (16.1)	6 (17.1)	48 (16.0)	
Number of ANC visits in current pregnancy	1	19 (5.7)	2 (5.7)	17 (5.7)	0.999
	2-3	162 (48.2)	17 (48.6)	145 (48.2)	
	≥ 4	155 (46.1)	16 (45.7)	139 (45.7)	
History of foetal macrosomia n=228	Yes	14 (6.1)	6 (20.7)	8 (4.0)	<0.001
	No	214 (93.9)	23 (79.3)	191 (96.0)	
GDM in previous pregnancy n=228	Yes	1 (0.4)	0 (0.0)	1 (0.5)	0.702
	No	227 (99.6)	29 (100)	198 (99.5)	
History of abortions n=228	Yes	50(21.9)	6 (20.7)	44 (22.1)	0.609
	No	178 (78.1)	23 (79.3)	155 (77.9)	
History of unexplained stillbirth n=228	Yes	4 (1.8)	0 (0.0)	4 (2.0)	0.505
	No	224 (98.2)	29 (100.0)	195 (98.0)	
History of Caesarean delivery n=228	Yes	26(11.4)	2 (5.7)	24 (12.1)	0.414
	No	202 (88.6)	27 (93.1)	175 (87.9)	
Mid-upper arm circumference (MUAC) in (cm)	Normal (<28)	149 (44.4)	13 (37.1)	136 (45.2)	0.365
	Overweight/Obese (≥28)	187 (55.7)	22 (62.9)	165 (54.8)	
Body Mass Index (BMI) in (kg/m^2^)	Normal (18.5 - 24.9)	90 (26.8)	9 (25.7)	81 (26.9)	0.009
	Overweight (25-29.9)	144 (42.9)	8 (22.9)	136 (45.2)	
	Obese (≥30)	102 (30.4)	18 (51.4)	84 (27.9)	
Chronic hypertension	Yes	4 (1.2)	2 (5.7)	2 (0.7)	0.009
	No	332 (98.8)	33 (94.3)	299 (99.3)	
Family history of diabetes mellitus	Yes	42 (12.5)	8 (22.9)	34 (11.3)	0.050
	No	294 (87.5)	27 (77.1)	267 (88.7)	

**Factors associated with gestational diabetes mellitus:** at multivariable analysis, having a previous history of foetal macrosomia in any of the previous pregnancies (aOR: 5.53, 95% C.I: 1.29-23.65, p= 0.021) and a family history of diabetes mellitus (aOR: 4.45, 95% C.I:1.48-13.34, p= 0.008) were associated with GDM among women attending the ANC clinic of MRRH as shown in Table 3.

**Table 3:** factors associated with gestational diabetes mellitus among pregnant women attending the antenatal care clinic (ANC) clinic at the Mbarara Regional Referral Hospital

Variables		GDM N=35 n (%)	cOR (95%CI)	p-value	aOR (95% C.I)	p-value
Age (years)	<20	8 (22.9)	1.0		1.0	
	20-34	26 (74.3)	0.37 (0.15-0.88)	0.026	0.34(0.08-1.52)	0.159
	>34	1 (2.9)	0.24 (0.03-2.12)	0.200	0.27(0.02-3.99)	0.338
Level of education	Tertiary	6 (17.1)	1.0		1.0	
	Secondary	14 (40.0)	2.03(0.75-5.50)	0.163	1.59(0.51-4.98)	0.423
	Primary	14 (40.0)	2.28(0.84-6.20)	0.105	2.68(0.84-8.53)	0.094
	Uneducated	1 (2.9)	5.22(0.47-58.08)	0.179	-	-
Gravidity	1	6 (17.1)	1.0		1.0	
	2 - 4	21 (60.0)	2.10 (0.82-5.38)	0.122	1.17(0.40-3.49)	0.772
	≥ 5	8 (22.9)	4.69(1.51-14.61)	0.008	0.49(0.07-3.59)	0.485
Body mass index	18.5 - 24.9	9 (25.7)	1.0		1.0	
	25 - 29.9	8 (22.9)	0.53(0.20-1.43)	0.209	0.66(0.21-2.07)	0.478
	≥30	18 (51.4)	1.92(0.82-4.54)	0.133	1.64(0.55-4.91)	0.378
Previous history of foetal macrosomia (n=228)	No	23 (79.3)	1.0		1.0	
	Yes	6 (20.7)	6.23(1.98-19.54)	0.002	5.53(1.29-23.65)	0.021
Chronic hypertension	No	33 (94.3)	1.0		1.0	
	Yes	2 (5.7)	9.06(1.24-66.46)	0.030	6.41(0.49-83.30)	0.155
Family history of diabetes	No	27 (77.1)	1.0		1.0	
	Yes	8 (22.9)	2.33 (0.98-5.53)	0.056	4.45(1.48-13.34)	0.008

## Discussion

In our study, we found that 10.2% of the pregnant women attending the antenatal care clinic at MRRH had GDM. Women who had had history of a macrosomic baby in any of their previous pregnancies and family history of diabetes mellitus among the first-degree relatives were more likely to have GDM.

The prevalence of GDM in our study is similar to the average global prevalence 10% [4]. Studies in sub-Saharan African countries of Nigeria, South Africa and Ghana have shown a similar prevalence of 8% to 9.3% [18-20]. These may be similar to our findings because these studies used the WHO-2013 diagnostic criteria for GDM, and similarly they were hospital-based studies. However, our prevalence is lower than in previous studies done in Uganda, which reported a prevalence of 15.6% to 30.3% [7,8]. The difference in prevalence may be attributed to the fact that the studies considered all pregnant women at any gestational age and used glycosylated haemoglobin (HbA1c) and random blood sugar as screening methods for GDM [7] while the other was in a private hospital and urban setting [8]. Other studies done in Cameroon and Tanzania reported higher GDM prevalence of 19.5% and 27.9% respectively [15,21]. The higher GDM prevalence in Tanzania could be because the study was also carried out in an urban setting. The study from Cameroon used International Association of the Diabetes and Pregnancy Study Groups (IADPSG) diagnostic criteria that has also been reported to increase the prevalence of GDM in most studies because IADPSG criteria does not have an upper limit for both FBS and OGTT levels for example using FBS ≥ 5.1 and 2 hour OGGT FBS 8.5 mml/l as compared to other diagnostic criteria [22].

Our study has a higher prevalence compared to what has been found in other studies from countries bordering Uganda. A prospective study done at three hospitals in Kenya found a prevalence of 2.9% [23] while a cross-sectional study in eight health centres in Dar-es-Salaam city in Tanzania, reported a prevalence of 5.9% [24] and in Rwanda at 8 public health centres, a prevalence of 3.2% was reported [25]. The low prevalence may be because these were multicentre studies and some of these studies used the 1999 WHO criteria for the diagnosis of GDM [24].

Our study showed that pregnant women with previous history of foetal macrosomia in any of their previous pregnancies were more likely to have GDM. This is similar to what was found in other studies [21,26]. Previous history of foetal macrosomia in the previous pregnancy may be an indicator of poor blood glucose control, undiagnosed GDM and maternal diet which may predispose the pregnant women to GDM in the present pregnancy [27,28]. Foetal macrosomia in GDM is mainly due to the increased insulin resistance in pregnant women with higher amount of blood glucose passing through the placenta into the foetal circulation. As a result, the extra glucose is stored in the foetus as body fat causing macrosomia [28].

Women with a family history of DM in the first-degree relatives were more likely to have GDM. This finding is consistent with findings from other studies done in Tanzania, Ethiopia and Peru [5,24,29,30]. This finding may be because of genetically inherited dysfunctional Beta-Cell genes (KCNJ11, KCNQ1, MTNR1B, IGF2BP2, rs7754840 and rs7756992 in CDKAL1) that cause insulin resistance and familial predisposition to insulin secretory defects passed on from one generation to another that cause hyperglycaemia. Such predisposition when coupled with insulin resistance in pregnancy may predispose women to GDM [31,32].

Our study had some strengths and limitations. We screened and tested pregnant women as at the point of care in the natural clinical setting. Although we relied on the participant´s report of when they last had a meal to ascertain their 8-hour fast, this may have a strength in integrating such a screening approach into routine clinical care as we offer glucose testing for women who present at the antenatal clinic. This may facilitate early diagnosis as some of the women may not report at the next visit. The birth weights of babies from the previous deliveries were self-reports from the participants with the potential for recall bias.

## Conclusion

One in every ten pregnant women attending the antenatal care clinic at Mbarara Regional Referral Hospital had GDM. There is need for routine screening for GDM among all women attending antenatal care at MRRH especially for women with history of foetal macrosomia in the previous pregnancies and a family history of diabetes mellitus in the first-degree relatives. These women should be specifically identified during the antenatal visits and should be screened for GDM. There is need for longitudinal studies to understand the prognostic and clinical implications of GDM including maternal and foetal mortality and morbidity in a rural setting such as South-Western Uganda.

### What is known about this topic

- *Gestational diabetes mellitus is a major cause of morbidity and mortality among pregnant women;*
- *However, there is variation in the burden of gestational diabetes mellitus across different settings as well as associated factors*.

### What this study adds

- *Our study shows that gestational diabetes mellitus is common among women attending antenatal care at a tertiary care facility in Uganda;*
- *Women with a history of delivering a macrosomic baby and those with a family history of diabetes mellitus were likely to have gestational diabetes mellitus*.

## References

[1] Farrar D, Duley L, Dowswell T, Lawlor DA (2017). Different strategies for diagnosing gestational diabetes to improve maternal and infant health. Cochrane Database Syst Rev.

[2] American Diabetes Association (2019). 2. Classification and Diagnosis of Diabetes: Standards of Medical Care in Diabetes-2019. Diabetes Care.

[3] World Health Organization (2013). Diagnostic Criteria and Classification of Hyperglycaemia First Detected in Pregnancy.

[4] Gyasi-Antwi P, Walker L, Moody C, Okyere S, Salt K, Anang L (2020). Global Prevalence of Gestational Diabetes Mellitus: A Systematic Review and Meta-Analysis. New American Journal of Medicine.

[5] Muche AA, Olayemi OO, Gete YK (2019). Prevalence and determinants of gestational diabetes mellitus in Africa based on the updated international diagnostic criteria: a systematic review and meta-analysis. Arch Public Health.

[6] Natamba BK, Namara AA, Nyirenda MJ (2019). Burden, risk factors and maternal and offspring outcomes of gestational diabetes mellitus (GDM) in sub-Saharan Africa (SSA): a systematic review and meta-analysis. BMC Pregnancy Childbirth.

[7] Kiiza F, Kayibanda D, Tumushabe P, Kyohairwe L, Atwine R, Kajabwangu R (2020). Frequency and Factors Associated with Hyperglycaemia First Detected during Pregnancy at Itojo General Hospital, South Western Uganda: A Cross-Sectional Study. J Diabetes Res.

[8] Nakabuye B, Bahendeka S, Byaruhanga R (2017). Prevalence of hyperglycaemia first detected during pregnancy and subsequent obstetric outcomes at St. Francis Hospital Nsambya. BMC Res Notes.

[9] Gorban de Lapertosa S, Alvariñas J, Elgart JF, Salzberg S, Gagliardino JJ, EduGest group (2020). The triad macrosomia, obesity, and hypertriglyceridemia in gestational diabetes. Diabetes Metab Res Rev.

[10] Roy R, Nguyen-Ngo C, Lappas M (2020). Short-chain fatty acids as novel therapeutics for gestational diabetes. J Mol Endocrinol.

[11] Roy I, Burande A, Choubey R (2019). Obstetric outcome in grand multipara-a Meghalaya experience. J OBGYN.

[12] American Diabetes Association (2020). 2 Classification and Diagnosis of Diabetes: Standards of Medical Care in Diabetes-2020. Diabetes Care.

[13] Azeez T (2020). Gestational Diabetes Mellitus: Unique Challenges in Sub-Saharan Africa. Journal of Gynecology Research Reviews & Reports SRC/JGRRR-127.

[14] Dea AG, Sullivan KM Open Source Statistics for Public Health.

[15] Mukuve A, Noorani M, Sendagire I, Mgonja M (2020). Magnitude of screening for gestational diabetes mellitus in an urban setting in Tanzania; a cross-sectional analytic study. BMC Pregnancy Childbirth.

[16] Johnson B (2020). 01: THE 2019 UK PHYSICAL ACTIVITY GUIDELINES.

[17] Food and Agriculture Organization of the United Nations (2016). Minimum Dietary Diversity for Women: A Guide to Measurement (FAO, Rome).

[18] Macaulay S, Ngobeni M, Dunger DB, Norris SA (2018). The prevalence of gestational diabetes mellitus amongst black South African women is a public health concern. Diabetes Res Clin Pract.

[19] Olagbuji BN, Atiba AS, Olofinbiyi BA, Akintayo AA, Awoleke JO, Ade-Ojo IP (2015). Prevalence of and risk factors for gestational diabetes using 1999, 2013 WHO and IADPSG criteria upon implementation of a universal one-step screening and diagnostic strategy in a sub-Saharan African population. Eur J Obstet Gynecol Reprod Biol.

[20] Oppong SA, Ntumy MY, Amoakoh-Coleman M, Ogum-Alangea D, Modey-Amoah E (2015). Gestational diabetes mellitus among women attending prenatal care at Korle-Bu Teaching Hospital, Accra, Ghana. Int J Gynaecol Obstet.

[21] Egbe TO, Tsaku ES, Tchounzou R, Ngowe MN (2018). Prevalence and risk factors of gestational diabetes mellitus in a population of pregnant women attending three health facilities in Limbe, Cameroon: a cross-sectional study. The Pan African Medical Journal.

[22] Akgöl E, Abusoglu S, Gün FD, Ünlü A (2017). Prevalence of gestational diabetes mellitus according to the different criterias. Turk J Obstet Gynecol.

[23] Pastakia SD, Njuguna B, Onyango BA, Washington S, Christoffersen-Deb A, Kosgei WK (2017). Prevalence of gestational diabetes mellitus based on various screening strategies in western Kenya: a prospective comparison of point of care diagnostic methods. BMC Pregnancy Childbirth.

[24] Mwanri AW, Kinabo J, Ramaiya K, Feskens EJ (2014). Prevalence of gestational diabetes mellitus in urban and rural Tanzania. Diabetes Res Clin Pract.

[25] Meharry PM, Tengera O, Rulisa S, Byambu AK, Nietert PJ, Byiringiro S (2019). Prevalence of gestational diabetes mellitus among women attending antenatal care at public health centers in Rwanda. Diabetes Res Clin Pract.

[26] Mwanri AW, Kinabo J, Ramaiya K, Feskens EJ (2015). Gestational diabetes mellitus in sub-Saharan Africa: systematic review and metaregression on prevalence and risk factors. Trop Med Int Health.

[27] MacNeill S, Dodds L, Hamilton DC, Armson BA, VandenHof M (2001). Rates and risk factors for recurrence of gestational diabetes. Diabetes Care.

[28] Kc K, Shakya S, Zhang H (2015). Gestational diabetes mellitus and macrosomia: a literature review. Ann Nutr Metab.

[29] Muche AA, Olayemi OO, Gete YK (2019). Prevalence of gestational diabetes mellitus and associated factors among women attending antenatal care at Gondar town public health facilities, Northwest Ethiopia. BMC Pregnancy Childbirth.

[30] Larrabure-Torrealva GT, Martinez S, Luque-Fernandez MA, Sanchez SE, Mascaro PA, Ingar H (2018). Prevalence and risk factors of gestational diabetes mellitus: findings from a universal screening feasibility program in Lima, Peru. BMC Pregnancy Childbirth.

[31] Guo F, Long W, Zhou W, Zhang B, Liu J, Yu B (2018). FTO, GCKR, CDKAL1 and CDKN2A/B gene polymorphisms and the risk of gestational diabetes mellitus: a meta-analysis. Arch Gynecol Obstet.

[32] Ehrmann DA, Sturis J, Byrne MM, Karrison T, Rosenfield RL, Polonsky KS (1995). Insulin secretory defects in polycystic ovary syndrome. Relationship to insulin sensitivity and family history of non-insulin-dependent diabetes mellitus. J Clin Invest.

